# Understanding the Inguinal Sinus in Sheep (*Ovis aries*)—Morphology, Secretion, and Expression of Progesterone, Estrogens, and Prolactin Receptors

**DOI:** 10.3390/ijms18071516

**Published:** 2017-07-13

**Authors:** Graça Alexandre-Pires, Catarina Martins, António M. Galvão, Margarida Miranda, Olga Silva, Dário Ligeiro, Telmo Nunes, Graça Ferreira-Dias

**Affiliations:** 1 CIISA-Faculty of Veterinary Medicine (FMV), Universidade de Lisboa, Av. Universidade Técnica, 1300-477 Lisboa, Portugal; gpires@fmv.ulisboa.pt; 2 CEDOC-Chronic Diseases Research Center, Immunology, NOVA Medical School, Universidade Nova de Lisboa, Rua Câmara Pestana n° 6, 6-A Edifício CEDOC II, 1150-082 Lisboa, Portugal; catarina.martins@fcm.unl.pt; 3 CIISA-Faculty of Veterinary Medicine (FMV), Universidade de Lisboa, Av. Universidade Técnica, 1300-477 Lisboa, Portugal; agalvao@fmv.ulisboa.pt; 4 iMed.ULisboa, Pharmacological and Regulatory Sciences Group, Faculty of Pharmacy, Universidade de Lisboa, Av. Gama Pinto, 1649-003 Lisbon, Portugal; mimiranda@live.com.pt (M.M.); odsilva@campus.ul.pt (O.S.); 5 Centro de Sangue e Transplantação de Lisboa, IPST,IP Alameda das Linhas de Torres 117, 1749-005 Lisbon, Portugal; dario@ipst.min-saude.pt; 6 Microscopy Center, Faculty of Sciences, Campo Grande, 1749-016 Lisboa, Portugal; telmonunes@hotmail.com; 7 CIISA-Faculty of Veterinary Medicine (FMV), Universidade de Lisboa, Av. Universidade Técnica, 1300-477 Lisboa, Portugal; gmlfdias@fmv.ulisboa.pt

**Keywords:** inguinal sinus, morphology, transcription, ESR1, ESR2, PGR, PRLR, chemical compounds, triterpenoids

## Abstract

Post-parturient behavior of mammalian females is essential for early parent–offspring contact. After delivery, lambs need to ingest colostrum for obtaining the related immunological protection, and early interactions between the mother and the lamb are crucial. Despite visual and auditory cues, olfactory cues are decisive in lamb orientation to the mammary gland. In sheep, the inguinal sinus is located bilaterally near the mammary gland as a skin pouch (IGS) that presents a gland that secretes a strong-smelling wax. Sheep IGS gland functions have many aspects under evaluation. The objective of the present study was to evaluate sheep IGS gland functional aspects and mRNA transcription and the protein expression of several hormone receptors, such as progesterone receptor (PGR), estrogen receptor 1 (ESR1), and 2 (ESR2) and prolactin receptor (PRLR) present. In addition, another aim was to achieve information about IGS ultrastructure and chemical compounds produced in this gland. All hormone receptors evaluated show expression in IGS during the estrous cycle (follicular/luteal phases), pregnancy, and the post-partum period. IGS secretion is rich in triterpenoids that totally differ from the surrounding skin. They might be essential substances for the development of an olfactory preference of newborns to their mothers.

## 1. Introduction

Shortly after birth, the mammalian neonate and its mother interact in order to favor parent–offspring contact. Under evolutionary pressure to meet the needs of their neonates, selected behaviors of post-parturient females occur, with nursing patterns being very broad across species concerning duration or frequency [1]. In fact, nursing patterns are different depending on the species. The amount of parental nursing and milk investment determines not only the number of young the parent can produce, but also affects the offspring’s fitness. As a an example, while penguins are known to sacrifice their own health and wellbeing in exchange for the survival of their young, Sprague–Dawley rats eject milk to feed their pups only when the mother is asleep and therefore without any nursing care burden [2,3].

In eutherian mammals, the mammary gland suffers modifications due to the presence of a prehensile nipple that facilitates the milk intake. The neonate, especially in a precocious mammal, is highly aroused by the stimulatory process caused by birth and is tuned towards the sensory cues that facilitate localization of the mother’s nipples. Since lambs are born with fully functional sensory systems, they can use multisensory cues to find the teat. In addition, ewes lick their lambs after birth and make a low-pitched moaning, which is an important behavior for the development of ewe–lamb binding and directs the lamb towards the inguinal region. That area is wool-free and has a higher temperature, eliciting lambs’ nosing and directing them towards IGS. When considering lambing, the desired outcome is to have more lambs born alive and allowing them to get adequate colostrum so they stay alive [4,5].

Newly born mammals have to reach the source of milk as promptly as possible to ensure uninterrupted mother-to-offspring transfer of hydration and nutrients. Colostrum intake guards against immediate exposure to micronutrients and antioxidants, passive immunization, innocuous bacterial strains, growth factors, and a range of bioactive peptides that control conservative behavioral function [4,5].

While some species give birth to altricial neonates, other species bear precocial or semiprecocial newborns whose behavior is under the control of all their senses (e.g., ungulates, some rodents). We do not fully understand how mammalian neonates perceive and analyze chemosignals and the olfactory scene that is associated with the mammary structure. The fact is that, by staying alive, the majority of them prove their competence to position themselves adequately from the very first exposure to a nipple. Suckling is the most intimate form of contact with the maternal body, which strengthens the relationship with the mother [6]. Besides maintaining the newborn’s warmth, nursing/suckling interactions also facilitate olfactory learning in newborns, as long as suckling and the olfactory stimulus temporally overlap [7].

Chemical communication plays a major role in mammalian behavior and starts by understanding olfactory communication and acquiring knowledge on body sources of odors and the behaviors associated with their deposition. This is the starting point for knowledge concerning putative chemosignals. Some insights concerning the mechanisms used by the newborn for detection and transduction of chemical stimuli are also needed. Odors refer to any chemical released by an individual, that is potentially detectable by another individual, and a pheromone is an odor that elicits a predictable and stereotypical behavior or physiological response, provides specific information, or modulates responses in the receiving individual [8]. Some of those chemosignals are of skin and gland origin (examples: the tarsal gland of the black-tailed deer, the inguinal and chin glands of rabbits for individual identification, the preputial glands in mature male pigs, and submaxillary glands for mating stance in estrus gilts) [9,10,11].

Across very different phylogenies, the mechanisms for pheromones and odor learning have much in common. The medial amygdala appears to be involved in both the recognition of social odors and their association with chemosensory information sensed by the vomeronasal system and sensory neurons and GABAergic interneurons in the olfactory bulb, which are continuously replaced. In fact, this contradicts the idea that neurogenesis is purely restorative in the adult stage since it develops the necessary plasticity for induction and maintenance of learned chemosensory responses [12,13].

In the ungulate family (*Caprinae*, *Cephalophinae*, *Antilopinae*), circumscribed scent glands are located bilaterally as pouches or pockets near the exterior base of the udder. Several morphological features that present a gland, such as the infraorbital sinus, the interdigital sinus, and the inguinal sinus, characterize the genus *Ovis aries*. Therefore, we can find the inguinal sinus in the ram as well as in the ewe. Different research groups have pointed out different responsibilities in the secretion of these three different glands. In some cases, they function as trail glands, while in others they have a role in the known “male effect”. By producing pheromones in the male, they elicit female reproductive physiologic responses [14,15].

After giving birth, mothers position their bodies so that the neonate finds the mammary zone. The suckling/nursing relationship becomes the core of their physiological and behavioral relationship and involves the blocking of the estrous cycle, the stimulation of lactation, and the development of a bond with repercussions for the future of the neonate, as this contact stimulates the mother’s care and the infant’s willing to find the nipple and ingest milk. Since lambs are born with fully functional sensory modalities, they can use multisensory cues to find the teat. In return, ewes lick their lambs after birth and present low-pitched bleating, an important behavior for the development of ewe–lamb attachment and direct the lamb towards the inguinal region, where areas free of wool have a higher temperature, eliciting the lambs’ nosing and directing them towards IGS. When considering lambing, the pressure is to have as many lambs born alive as possible and allow them to get adequate colostrum so that they stay alive. The parental investment in producing a new young life (or two) per reproductive cycle is very high and the behavior of the young and its likelihood to survive is under the responsibility of its mother at the beginning of its life throughout lactation. This mother–young unit is a major part of the welfare of the neonate and inadequate maternal care invariably leads to early death [6,16].

In the literature, references to inguinal gland wax production describe a strong-smelling substance that seems to activate udder-seeking behavior, in combination with vocal and tactile stimuli [17,18]. Records of respiration and heart rates demonstrated that non-suckling lambs respond to the smell of mother’s inguinal sinus production in a more reactive way than the response found when odors of wool or milk from an unfamiliar ewe are available. In addition, newborns can discriminate between the smell of their mothers and that of an alien ewe and it was shown that when lambs are made anosmic by applying lidocaine to their nostrils, localization of the teat was delayed [19,20,21]. Initiation of suckling is dependent on variable blends of maternal “signature odors” that are learned and recognized prior to first suckling. In rabbits, the pheromone responsible for initiating suckling has already been identified and newborn mice require maternal olfactory or “signature odors” cues to start suckling since this blend of volatile odors (not necessarily a classical pheromone) produced from the mother elicits the behavior [22]. In sheep, maternal behavior (low-pitched bleats, licking, and nursing) is triggered by changes of plasma progesterone and estradiol around parturition, and the release of oxytocin in the brain [17]. Thus, the hypothesis we presented was that sheep IGS might play a role as a chemosensory clue to the newborn lamb.

As IGS gland functions are in many aspects under evaluation, the objective of the present study was to evaluate: (i) IGS morphology, histology and ultrastructure; (ii) mRNA transcription and protein expression of progesterone (PGR), estrogen receptors 1 (ESR1) and 2 (ESR2) and prolactin receptor (PRLR). Also, as these hormones show associations with sexual and nursing behavior in many aspects, achieving information about putative changes in the chemical compounds produced in this body sinus along the estrous cycle was also one of the goals.

## 2. Results

**Figure 1:**
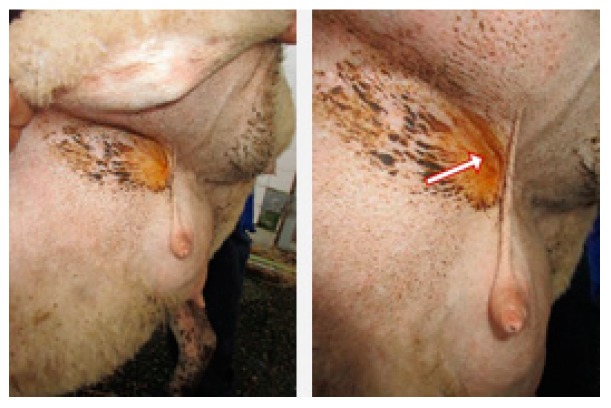
By abduction of the hind-left leg one can observe the skin pouch (IGS) over the mammary gland. On the right, the arrow points out the IGS and its yellowish secretion.

**Figure 2:**
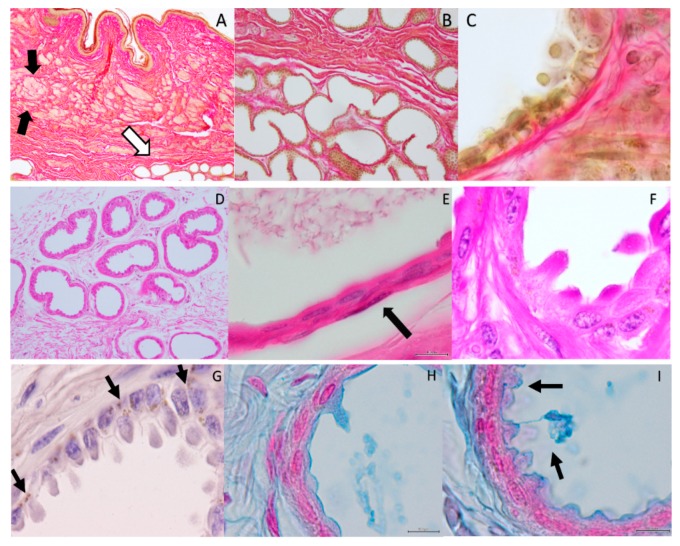
Histology-Bar = 100 µm. *Van Gieson’s* Stain. (**A**) Organization of the inguinal sinus presenting sebaceous (black arrows) and acinar glandular fields (white arrow); (**B**) collagen fibers sustain the secretory epithelium of the acinar glands that presents; (**C**) cellular protrusions towards the glandular lumen (apocrine secretion). Parenchyma rich in glycogen can be observed and its amount varies in different areas of the gland being the secretory cells in different stages of secretion production in different areas of the gland–PAS; (**D**–**F**); In (**E**) the arrow points out a myoepithelial cell. (**D**) = 40× magnification; (**E**) = 1000×. Visualizations of lipid granules with Sudan black stain; (**G**) Alcian blue stain depicts mucin content; (**H**,**I**) Magnification = 1000×.

Near the mammary gland, one can observe by abduction of the hind limb the inguinal sinus located bilaterally as pockets at the external base of this gland. When exposed, a yellowish substance with a wax appearance seems to spread downward to the teats (Figure 1). The histology of the inguinal sinus demonstrates invaginated skin presenting sebaceous and acinar glandular fields, with collagen sheath fibers sustaining the secretory epithelium (Figure 2A–C). The acinar glands appear with different patterns of secretion, from a resting phase to the development of huge cellular protrusions of apocrine secretions towards the glandular lumen (for example comparing Figure 2C,F,G,H). According to dye affinities, the parenchyma is shown to be rich in glycogen (PAS; Figure 2E,F) and lipid granules (Sudan Black stain, 2 G), and mucin content as well (Alcian blue-2H-I). Myoepithelial cells can be depicted (Figure 2E). Secretion production appears with an uneven distribution in different areas of the inguinal pouch and in different acinar units, as shown in different plates of Figure 2.

**Figure 3:**
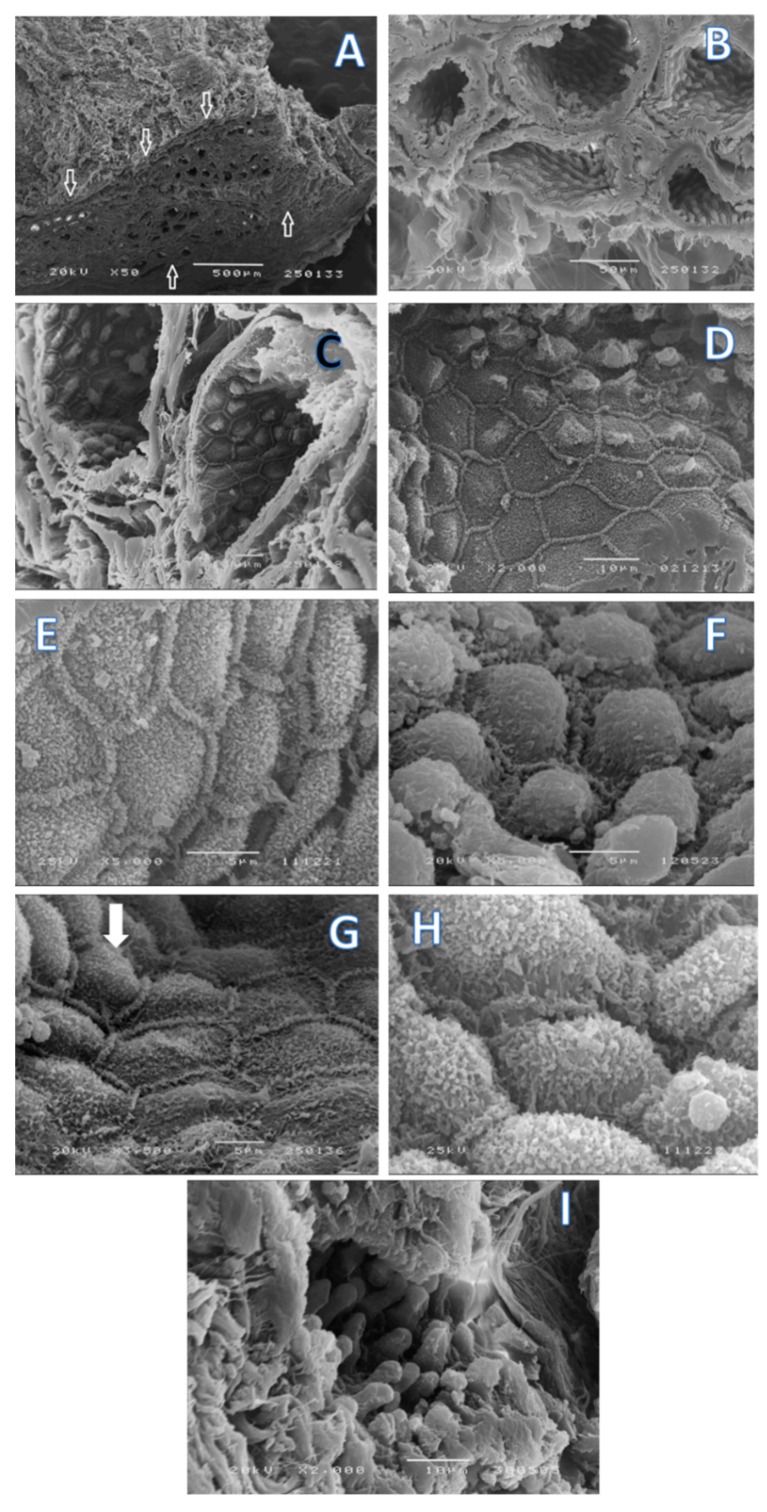
Scanning electronic images of IGS. In (**A**) (bar = 500 µm); (**B**) (bar = 50 µm) and (**C**) (bar = 10 µm) it is clear that apocrine glandular structures appear in clusters inside the IGS. Luminal surface can show a paved appearance; (**D**) bar = 10 µm or an irregular one resulting from the secretory process. Secretory cells appear in different stages of differentiation, where fragments of secretion are being “pinched off” exhibiting secretory vesicles (secretion blebs), while cells maintain a clear demarcation with neighboring cells by means of rows of microvilli; (**D**) (bar = 10 µm); (**E**) (bar = 5 µm) and (**F**) (bar = 5 µm). A progressive gland filling process results on the upsurge of bulge-like structure; (**G**) (bar = 5 µm) and (**H**) (bar = 5 µm). Some cells appear to be in a transitional process where demarcation with surrounding cells is no longer as visible resulting from the development of apical protrusions that in a final stage denote a smooth plasma membrane devoid of microvilli and covering the protrusions; (**I**) bar = 10 µm.

Ultrastructural observations of scanning electronic microscopy (SEM) (Figure 3) showed that the apocrine glandular units are of alveolar type but not tubuloalveolar and that these secretion units appear in clusters (Figure 3A,B). Luminal surface secretion presents a paved appearance, with secretory cells in diverse stages of differentiation (Figure 3C). In fact, some acini at different stages depict fragments of secretion being “pinched off”, exhibiting secretory vesicles or secretion blebs (Figure 3D,E), although cells preserve a clear demarcation with neighboring cells by means of rows of microvilli. Apical end-pieces show a progressive filling process that upsurges as bulge-like structures. In other acini, cells appear to be in a transitional process where the clear demarcation with surrounding cells is no longer as visible, resulting from the development of apical protrusions that in a final stage denote a smooth plasma membrane devoid of microvilli and covering the protrusions (Figure 3F,H).

**Figure 4:**
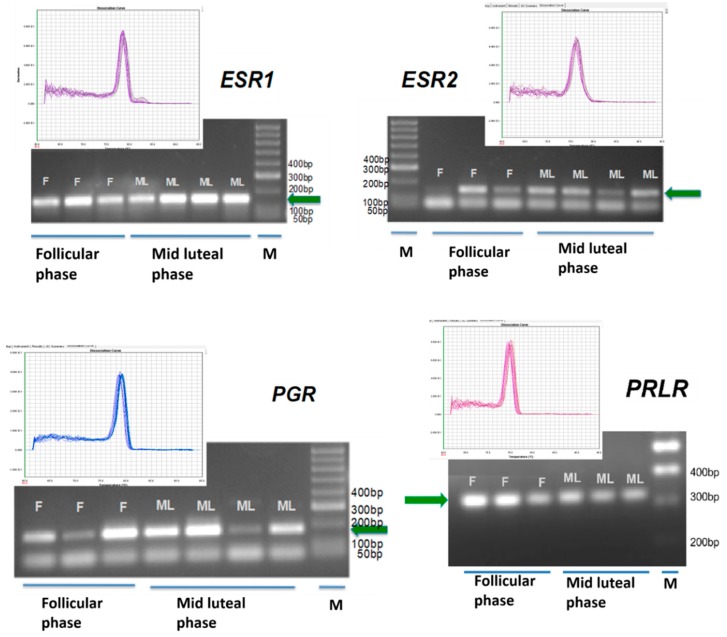
Qualitative PCR electrophoresis gel and dissociation curves of real-time PCR confirming estrogen receptor 1 (*ESR1*), and 2 *ESR2*, progesterone receptor (*PGR*) and prolactin receptor (*PRLR*) gene transcription in the IGS in different phase of the estrous cycle. Green arrow indicates the specific gene band. (F) follicular phase; (ML) mid luteal phase; (M) DNA marker; bp (base pairs). All primers validated for 80 nM in the real time PCR run. Single product confirmation with the single peak in the dissociation curve.

According to specific primer sequences used for quantitative real-time PCR (reported in Section 4), it was possible to show transcription of mRNA for *ESR1*, *ESR2*, *PGR* and *PRL*R during the follicular and mid-luteal phase of the estrous cycle (Figure 4).

**Figure 5:**
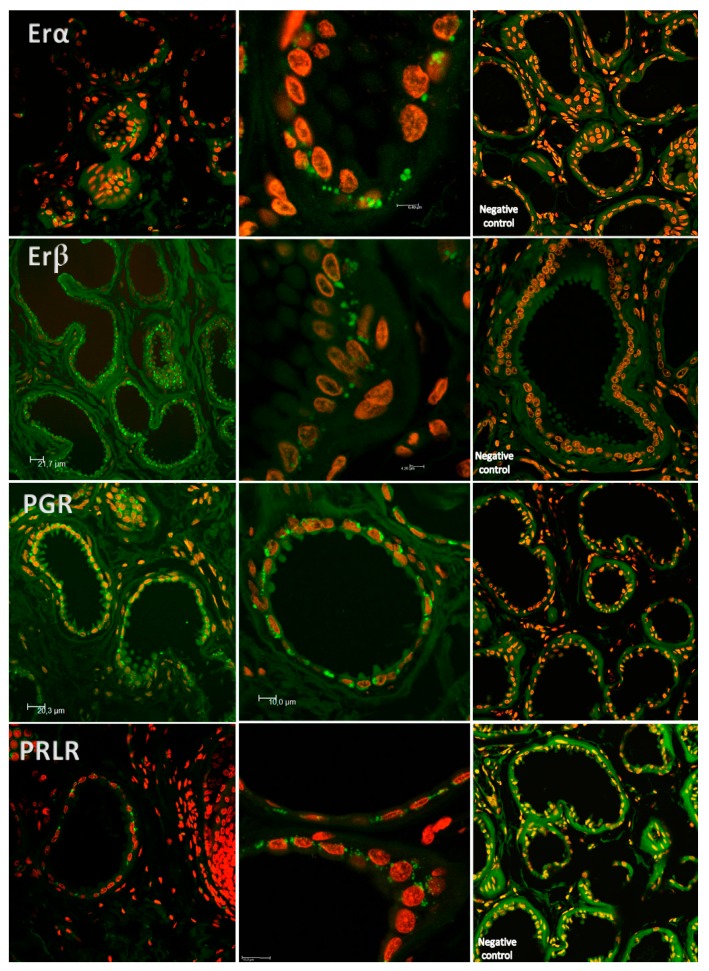
Examples of laser-scanning confocal fluorescence (LSC—lens 63.03 oil) images of IGS. One can observe immunoreactivity towards ESR1, ESR2, PGR and PRLR in cells of the apocrine glands labeled with PE and stained for the different receptors (fluorescence in green). Use of To-Pro-3 iodide for nuclear counterstaining (fluorescence in red).

Confocal scanning microscopy demonstrated immunoreactivity towards ESR1, ESR2, PGR and PLRL receptors regardless of the estrous phase or the differentiation of acini cells concerning secretion process. Evidence was found of non-nuclear estrogen receptor and PGR in a clear border basilar position (Figure 5).

**Figure 6:**
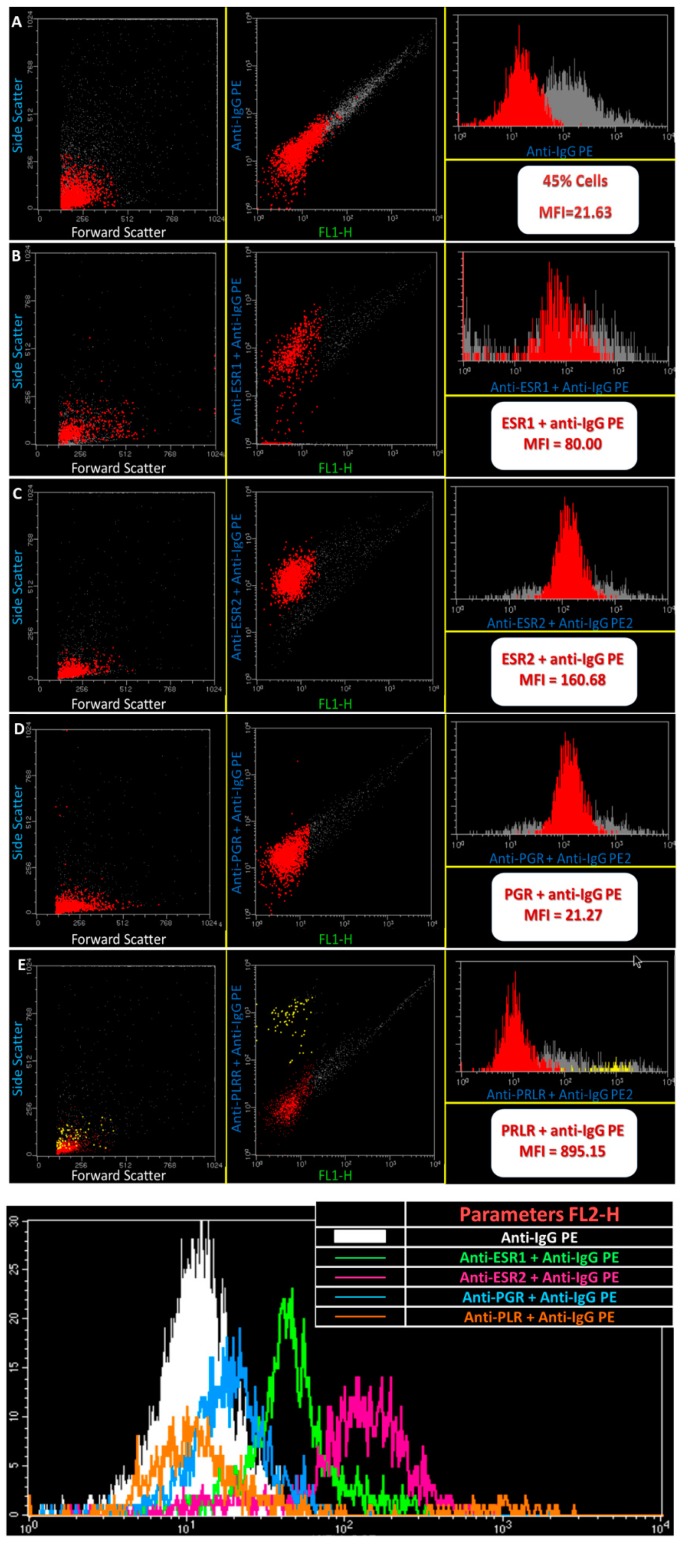
Flow cytometry analysis of gated cells of IGS. Examples of gated and dot plots and histograms showing the expression of ESR1, ESR2, PLRL and PGR. Shown flow cytometry data depict a positive cell expression towards the receptors under evaluation.

Flow cytometry analysis of cell suspensions showed cells with distinct auto fluorescence levels and different behavior towards ESR1, ESR2, PRLR and PGR); (Figure 6). Along with the different estrous cycle phases studied, PRLR and ESR2 positive cell populations showed always a higher fluorescence intensity compared to PGR and ESR1 positive cells (*p* < 0.05). At pregnancy, PRLR also showed a higher expression (*p* < 0.01) in comparison with other fluorescence intensities.

**Figure 7:**
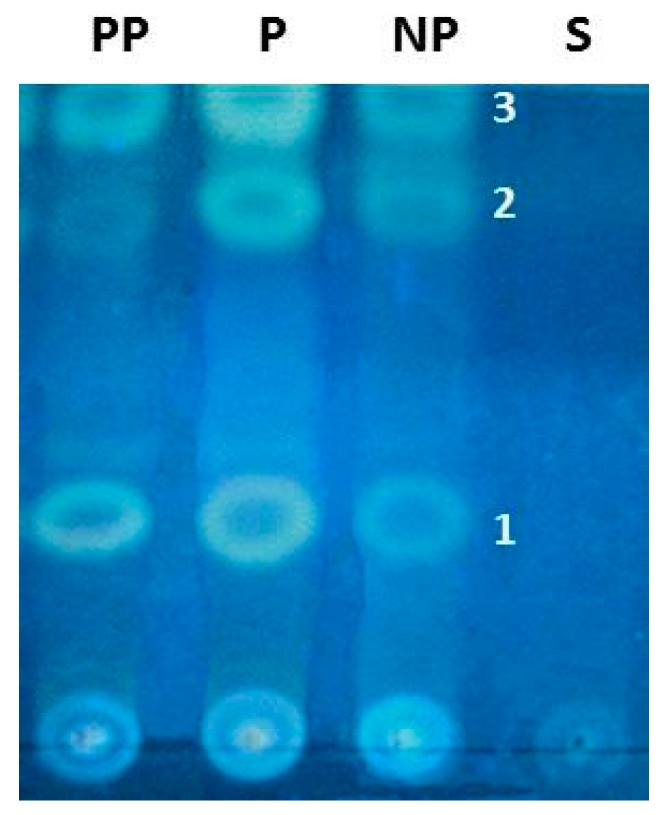
Chromatogram analysis revealed the presence of triterpenoids as marker chemical classes of post-partum ewes (PP), pregnant ewes (P) and non-pregnant (NP) samples, and a chemical profile that clearly portrait three major bands: 1, 2 and 3. These compounds were absent on S, the surrounding skin sample used as a negative control. The background color of the chromatogram is not uniform, which is a normal and recognized situation and does not interfere with the interpretation of the results.

Thin-layer chromatography was performed on the available set of samples (*n* = 28), applied semi-quantitatively. The conditions allowed for the identification of a chromatographic profile (retention factor, fluorescence) characteristic of the presence of triterpenoids, with three major bands (Figure 7, compounds 1–3) in post-partum ewes (PP), non-pregnant (NP), and pregnant ewes IGS (P). The relative intensities of these three bands in these groups of samples made it possible to infer the existence of a higher relative content of these triterpenoids in P samples, as also demonstrated in Supplementary Data.

## 3. Discussion

Several studies suggest that mammalian species have evolved multiple strategies to release olfactory signals to their offspring and ensure the onset of suckling as a critical behavior [23]. These tactics can be a pheromone-mediated behavior, or a reply to signature odors. Both appear to elicit innate behavior [22]. In the domestic pig, for example, after washing the sow’s abdomen with organic solvents, the piglets lost teat localization [24]. As reported, mammalian females are known to use odor cues to control infant state, attention and directional responses, to delay distress responses, to stimulate breathing and positive oral actions, and finally they can boost learning. Female–offspring odor communication in European rabbits and humans is representatives of evolutionary extremes in terms of the structure and dynamics of mother–infant relationships, and levels of neonatal autonomy. In fact, both species have evolved mammary structures and chemosignal sources under greasy fixatives [24]. These features confer on them a chemo-communicative function and promote the success of the offspring’s approach and exploration of the maternal body surface, resulting in effective initial feeds and rapid learning of maternal identity [25]. In women, for example, the literature reports several volatile compounds in the nipple/areola region during pregnancy and after childbirth that are not present in other phases [26]. Human neonatal reactivity to these areolar odors tested against several reference stimuli (e.g., human milk or sebum, solvent, vanilla, fresh cow’s milk, cow’s-milk-based formula) showed that pure Montgomery gland secretion elicits more orofacial activity. Nevertheless, further investigation is necessary to pin down the volatile compounds that can then be evaluated under the nose of human newborns in repeatable bioassays. So far, no evidence for any chemo-stimulus that would qualify as a pheromone is at hand in primates, including human mother-to-infant communication [23,27].

Lactating rabbits emit in their milk a volatile aldehyde, 2-methylbut-2-enal, that provokes searching and grasping behaviors in neonates [28,29]; newborn rabbits’ survival depends on the perception of this odor signal emitted from the mother’s *ventrum* area that allows them to locate the mother’s nipples and suckle. Emission of nipple pheromones in the rabbit is induced during pregnancy by the combined action of estrogen and progesterone [11,30].

It was recently demonstrated that peripheral olfactory neurogenesis driven by estrogen occurs in the vomeronasal organ. A fraction of those cells are able to extend their dendrites to contact the vomeronasal lumen and detect proteins that show pheromone activity, while concomitant differences in gene expression in the vomeronasal transcriptomes of pregnant female mice also occur [11]. In sheep, endocrine changes at parturition, together with interactions with the newborn, modulate cell proliferation and neurogenesis in the sub-ventricular zone, the main olfactory bulb, and the dentate gyrus. Also at parturition, with or without interactions with the lamb for two days, a downregulation of the number of newly born cells in those neurological areas occurs in comparison to non-pregnant sheep. Therefore, in sheep it was postulated that the downregulation of cell proliferation observed in the early post-partum period could facilitate the olfactory perceptual and memory demands associated with maternal behavior, by favoring the survival and integration of neurons born earlier [31]. This situation contrasts with the occurrence of peripheral olfactory neurogenesis driven by estrogen and prolactin, which induce an increase in neural progenitors in the sub-ventricular zone of the lateral ventricle of the brain in other species [13]. A huge field of possibilities must be tested since these facts suggest the likelihood that lamb post-partum survival might depend on an olfactory perception fulfilled by the production of substances present in the IGS of the mother.

A common factor that characterizes most post-partum lamb deaths is the disturbance of bond formation between ewes and their offspring [32]. The glandular area near the mammary gland produces a substance with a yellowish aspect that spreads down the teats. This glandular substrate can dislodge itself while the animal walks or licks the area, making lambs reactive when exposed to it by means of directional movements of the head resulting in an increase in respiratory and cardiac rates [33]. Moreover, the odor of the IGS secretion elicits a more major response than the odor of wool or milk. It should be underlined that the maternal “wax production” by the IGS is more reactogenic than a similar product from an alien ewe. Moreover, newly born lambs showed a reaction to ovine milk odor even if impregnated in a cloth, a situation that did not occur when a scentless and humid cloth was used instead, and it was shown that when lambs are made anosmic by having lidocaine applied to their nostrils, localization of the teat was delayed [1]. Altogether, data from different researchers call attention to IGS production as a potential candidate for a strong scent effect. Although no studies have pointed out the exact constitutive compounds of IGS, our data have shown that when comparing IGS secretions it is clear that they show differences compared to the surrounding skin secretions and change from early motherhood. Although it was outside the scope of this work, by looking at the possible neurogenesis associated with olfactory neurogenic repercussion of this IGS secreted substance or how this complex material will behave under down/upregulation of steroid hormones or prolactin, we gained an overall picture of the complexity of the mechanisms involved, considering the putative hormonal influence in olfactory signaling . As a unique feature, not present in the majority of other animals, evaluation of IGS and its production in our work aimed to find out their particularities with respect to normal skin and the nature of this substance in different reproductive phases of the ewe life cycle (estrous cycle, pregnancy and post-partum). The differences we have found might have an impact on sheep herd management.

Thin-layer chromatography (TLC) is a method for the chemical screening of compounds, considered one of the first steps to establishment of the chemical profile of a sample. The thin-layer chromatography conditions used allowed us to identify a chromatographic profile characteristic of triterpenoids [34] present on PP, NP and P. The relative intensities of these three bands on these groups of samples made it possible to infer the existence of a higher relative content of these triterpenoids in P samples. This technique was applied semi-quantitatively to the available samples. It is expected that after structural identification of the three detectable marker compounds, they will be quantified by means of a suitable method specifically validated for that purpose. Nevertheless, the NP group’s lowest relative content on these compounds calls attention to their putative involvement in chemo-communication and, as mentioned, further studies will be carried out in order to confirm the involvement of those triterpenoids in the development of an olfactory preference of the newborn lambs towards the mother’s mammary gland.

In sheep and goats the beginning of maternal behavior at parturition is under the control of hormonal changes and fetus expulsion [35,36]. Thus, our interest concerning the presence of progesterone, estrogens, and prolactin receptors in IGS in the ewe is justified beyond a down- or upregulation involved in peripheral olfactory neurogenesis, previously discussed. Prolactin (PRL) is a versatile hormone in mammals with effects in reproductive, sexual, metabolic, and immune functions, among others [37]. The distribution of PRLR and the awareness of several extra-pituitary PRL-expressing tissues, has called attention to the range of PRL actions beyond mammary gland function [38,39,40]. Rabbit maternal behavior consists of building an underlayer of fur during late pregnancy and displaying, with circadian periodicity, a single 3-min nursing bout/day across lactation. It is synthesized in multiple tissues and its biological actions are not limited solely to reproduction, as it has been shown to control a variety of behaviors [41]. Synthesis of female-attracting pheromones in amphibians is regulated by prolactin (PRL) and the responsiveness of the female vomeronasal epithelium is enhanced by PRL and estrogen [42]. Estrogen, androgen, progesterone, and prolactin regulate specific aspects of nest-building and promote the onset of maternal responsiveness. However, the maintenance of this behavior relies on stimuli from the litter. By preventing mother/young contact at parturition or during early lactation, maternal responsiveness changes or became abolished. In the rabbit, brain areas controlling the expression of nest- building and nursing were under investigation by implanting estradiol and locating the distribution of estrogen and prolactin receptors in the forebrain [43]. That work showed that ESR1 present in the preoptic region may mediate the stimulation of nest-building by estradiol and that prolactin-binding sites, located mainly in periventricular structures, are more abundant in late pregnancy and early lactation [34]. Moreover, in the rabbit, scent emission is responsible for nipple search and is depressed following ovariectomy but further stimulated by estradiol administration [43].

These aspects legitimate our findings of ESR1 and ESR2 receptors in the IGS, as these estrogen receptors might be involved in the process of signaling pathways that may regulate conspecific chemical messages attributed to IGS as binding steroid hormones to specific receptors. Therefore, generating changes in the rates of nucleic acids and proteins synthesis might result in chemosensation. We should underline that there are subtypes of cytoplasmic estrogen receptors, as they perform their biological actions in the cytosol at a fast rate to be compatible with transcriptional mechanisms [44,45,46,47,48,49,50,51,52]. The canonical model for ER-mediated regulation of gene expression involves the direct binding of dimeric ER to DNA sequences known as estrogen response elements, which are specific and inverted palindromic sequences [53]. ER can associate indirectly with promoters through protein–protein interactions with other DNA-binding transcription factors and interaction of ERs with E2 leads to transcriptional activation of the associated genes via the recruitment of coactivators and components of the basal transcriptional machinery [54,55]. The “genomic action” of steroid hormones occurs after a time lag of at least 2 h after E2 stimulation and explains some of the hormone functions in physiological and pathological situations. However, some effects are too rapid to account for genomic action [56]. In fact, cytosol receptors can be turned on and off, suggesting different roles in physiological functions and pathogenesis [51,52]. These findings point out the evidence for an important role of the non-nuclear estrogen receptor in a fast non-transcriptional response of cells to estrogen. The way they contribute to the signaling of the mammary gland has been ascertained and already demonstrated in the interdigital sinus of the ewe as well [57].

In the present study, we demonstrated that the IGS appears as an invaginated skin fold presenting sebaceous and acinar glandular areas sustained by collagen sheath fibers. Since the apocrine unit is alveolar, but not tubuloalveolar, the spread of the wax is probably a result of the compression of the medial face of the hind limb against the upper area of the mammary gland where the IGS is located. In addition, myoepithelial cells contribute to the expelling of the apocrine secretion toward the lumen of the acinar units. Acinar cells present lipid granules, which we should link with the greasy aspect of the IGS production. Mucin content is present and seen in the matrix. It might eventually contribute to the capacity to resist proteolysis to maintain the “scent” production characteristics of this gland for a longer period.

According to our data, we can pinpoint the same acini cells where exocytosis of the glandular content release occurs via a mechanism of non-protrusion [58,59,60]. In addition, a process of gradual accumulation of secretory products that form balloon-like swellings protruding into the lumen was also depicted [61]. In accordance with the presence of a mixed population of epithelial cells in the acini, a dual pattern secretion can result in the same alveolar unit, as demonstrated in the sweat glands of Karagouniko sheep [62], although the signaling pathway that triggers vesicular sorting in still under discussion [60,61,62,63]. The parenchyma is rich in glycogen, whose amount varies in different areas of the gland.

PRL simultaneously embraces a high diversity of physiological actions beyond mammary gland development or milk production. It is synthesized in multiple tissues and its biological actions are not limited solely to reproduction, as it has been shown to control a variety of behaviors [41,42]. This hormone appears in the background of pathological skin conditions and skin derivatives such as disruption of time regulation of hair growth cycles in mice [64,65], alopecia, and psoriasis [40].

Indeed, this fact raises questions about the primitive function of PRL. This would explain its maintenance during pre-mammalian evolution, as some of those functions attributed to PRL are associated with the post-mating phase of reproductive cycles in different reproductive strategies. These fit with seasonal gonadal suppression or behavioral changes, such as inhibition of aggression [66,67]. Seeing as the integument and its appendages (feathers, hair, glands, and the mammary gland itself) are the focal point of numerous PRL actions, even in non-mammalian vertebrates [40,68,69,70,71], and as it has already been revealed that PRL controls lobule-alveolar proliferation and differentiation of secretory epithelium [72,73], it is important to find its receptor’s presence in the IGS. Moreover, since PRL shares the signal transduction pathway used by a variety of cytokines and growth factors [74,75], this hormone might therefore be important in signaling the onset of lactation for lambs. Epithelial growth is known to result from combined effects of P_4_ and PRL, both triggering a juxtacrine RANKL signal, which induces alveolar growth [76,77,78,79,80]. The simultaneous presence of PGR and PRLR in the inguinal sinus gland can also suggest the role of PG and PRL in IGS function in sheep. Synthesis of female-attracting pheromones in some species is regulated by prolactin (PRL) and responsiveness of the female vomeronasal epithelium is enhanced by PRL and estrogen [42]. Overall, the evaluation of the chosen receptors under research in our study seems to be of interest since these receptors are needed for the related hormone action, which may work together in the putative mechanisms involved in IGS function.

Data found in the present work agree with previous reports and contribute to the body of knowledge. At this point, it is not possible to state that there is a direct cause/effect action between plasma E_2_, P_4_ and prolactin on IGS function. Nevertheless, the receptors of these hormones found in IGS present variations in the different phases of the estrous cycle and pregnancy. Thus, we do suggest the involvement of those hormones in IGS function in ewes. Their presence constitutes a hallmark and an important point to start to manipulate responses [81]. Further studies investigating these hormones’ regulation will be mandatory since estrogen and prolactin up- or downregulation behave differently among species when considering the generation of neural progenitors in the brain, as mentioned earlier. By widening this field of research, which will contribute to an understanding and further development of odor-specific products, we may be able to promote newborn lambs’ survival.

## 4. Materials and Methods

For the present work, IGS (*n* = 92) were collected post mortem from adult merino ewes for different evaluations, as described below. As the reproductive history of the ewes was unknown, their estrous cycle phases were determined based on ovarian structures and plasma progesterone (P_4_) concentrations. Therefore, when a pre-ovulatory follicle was present in the ovary, in the absence of a corpus luteum (CL), and plasma P_4_ concentration was below 1 ng/mL, the ewes were considered as being in the follicular phase. Nevertheless, the presence of a CL in the ovary, and plasma P_4_ concentration above 1 ng/mL, indicated the ewe was in the luteal phase. Right after collection, IGS samples were immersed in (i) RNAlater (AM7020, Ambion, Applied Biosystems, Carlsbad, CA, USA) for mRNA transcription quantification; (ii) 4% buffered formaldehyde, for histology, immunohistochemistry, and confocal microscopy; (iii) Karnovsky’s solution for ultrastructure studies; or (iv) a sterile tube with RPMI 1640 (Gibco-Brl, Gaithersburg, MD, USA) for flow cytometry studies. In addition, the contents of IGS were collected into sterile tubes for biochemical quantifications. Blood samples were drawn into heparinized tubes at the time of exsanguination (MonovettesVR-Sarstedt, Numbrecht, Germany) for further estrous cycle confirmation. Furthermore, from 28 sheep (*n* = 8 from follicular phase, *n* = 10 pregnant, and *n* = 10 post-partum period (1–3 days after delivery)), the content of IGS obtained post mortem as a byproduct from animals used for other research purposes was collected to evaluate putative variations in secretions.

Competent veterinary authorities monitored the experiments. The ethical committee of the Faculty of Veterinary Medicine (Lisbon, Portugal) approved these. Several authors are holders of Federation of European Laboratory Animal Science Associations (FELASA) grade C certificate, which permits designing and conducting laboratory animal experimentation in the European Union.

### 4.1. Histology Evaluation

Ovine IGS samples (follicular phase, *n* = 5; luteal phase, *n* = 5) were cut into small pieces, fixed in buffered formaldehyde for 24 h, and processed for light microscopic study. Tissue serial sections were cut (5 mM thick—Microtome Leica SM2000R, Berlin, Germany) and stained with Weigert Van Gieson for collagen detection, Periodic Acid Schiff to assess glycogen content, Alcian Blue for mucin detection, and Black Sudan for detection of lipid production [82].

### 4.2. Scanning Electron Microscopy

Scanning electron microscopy (SEM) evaluation of intact IGS tissue (follicular phase, *n* = 5; luteal phase, *n* = 5) was performed. Immersion of the intact IGS tissue in Karnovsky’s solution (Sigma-Aldrich, Lisboa, Portugal), rinsed in cacodylate buffer, and post-fixed in a 2% osmium tetroxide solution for 1h. Rinsed once again with cacodylate buffer and subsequently dehydrated in a graded ethanol series. Samples dried using the critical point drying method and coated with gold palladium. IS were mounted on stubs, observed in a scanning electronic microscope (JEOL 5200-LV, Tokyo, Japan), and photographed.

### 4.3. Flow Cytometry Analysis

Flow cytometry analysis of IGS was carried out to quantify the expression of ESR1, ESR2, PGR and PRLR proteins. Ewe IGS (estrus *n* = 14; diestrus *n* = 14) were removed with a surgical blade and collected in a sterile tube with 1 mL of RPMI 1640 (Gibco-Brl). After disaggregation of the tissue with a surgical blade, samples of whole IGS were centrifuged at 190 g for 10 min. Then, they were resuspended in phosphate-buffered saline solution (PBS-P3813 Sigma). Fixation and permeabilization of cell suspensions with FIX & PERM VR-Fixation and Permeabilization Kit (Invitrogen Laboratories, Life Technologies, Waltham, MA, USA) were performed for 15 min longer in the dark at room temperature. After a final washing step, the pellet was suspended once again in 500 mL of BD FACS Flow for no longer than 15 min in the dark, at room temperature. After a new washing and centrifugation step, RPE-conjugated secondary antibody (10 mL) was added and cells were incubated 15 min longer in the dark at room temperature. A final washing step was necessary, and the resulting pellet was suspended once again in 500 μL of BD FACS Flow (BD Biosciences, San Jose, CA, USA). Cell acquisition was performed on a BD FACS Calibur flow cytometer (BD Biosciences) and data were analyzed using Paint-A-Gate Pro and Cell-Quest Pro software (BD Biosciences). In each experiment, incubation of cells was done according to the above protocol but with the secondary antibody only. This control tube was performed in order to assess the level of unspecific fluorescence signal of the secondary antibody. Selection of primary antibodies’ dilutions was as follows: 1. Mouse monoclonal anti progesterone receptor (77201704 AbD Serotec, Kidlington, UK), diluted at 1:10 in PBS; 2. Mouse anti-human monoclonal antibody ESR1 (ref. 41700, Invitrogen, Dorset, UK), diluted at 1:10 in PBS; 3. Mouse anti-human polyclonal ESR2 (MCA2279S, AbD Serotec), diluted at 1:10 in PBS, 4. Mouse monoclonal (U5) to prolactin receptor (abcam 2772, Cambridge, UK), diluted at 1:10 in PBS. The secondary antibody used was R-phycoerythrin F(ab’)2 frag. of goat anti-mouse (F2653 Sigma).

### 4.4. Laser-Scanning Confocal Microscopy

The locations of ESR1, ESR2, PGR and PRLR protein in IGS were assessed using laser-scanning confocal microscopy (Leica TCS SP2, Leica Microsystems; Berlin, Germany). The same antibodies used in flow cytometry evaluation were employed in this study (*n* = 6). Incubation of antibodies was performed overnight with the following dilutions: (1) Mouse monoclonal anti-progesterone receptor diluted at 1:50; (2) estrogen receptor (ESR1) diluted at 1:50 and rabbit anti-human estrogen receptor (ESR2) diluted at 1:50. Again, the FIX & PERM VR Fixation and Permeabilization Kit (Invitrogen Laboratories, Life Technologies, Waltham, MA, USA) was used. Briefly, reagent A was added for no longer than 15 min in the dark at room temperature before the addition of the primary antibody, which was incubated for an hour. For another 15 min, solution B was added followed by the addition of the second antibody.

To-Pro-3 iodide 1 mM solution (Invitrogen Molecular Probes, Eugene, OR, USA) was used for nuclear counterstaining. Negative controls were performed by replacing the primary antibody with either rabbit polyclonal IgG (ab27478, Abcam), for antibodies developed in a rabbit, or mouse IgG (550878, BD Biosciences, San Jose, CA, USA) for antibodies developed in a mouse, with the same dilution and incubation times as the primary antibody, followed by To-Pro-3 iodide for nuclear counterstaining. Selected sections were photographed with confocal laser microscopy, Leica TCS SP2.

### 4.5. Genomic Analysis

Conventional polymerase chain reaction (PCR) was used to assess mRNA gene expression of *PGR*, *ESR1*, *ESR1* and *PRLR* in sheep’s inguinal glands (follicular phase, *n* = 5; luteal phase, *n* = 5) Specific primers for *PGR*, *ESR1*, *ESR2* and *PRLR* were designed (Table 1), as follows:

RNA was extracted from IGS tissue (Qiagen’s Kit for Total RNA Extraction and Purification; ref. 28704, Qiagen, Hilden, Germany) and DNA digested (RNase-free DNase Set; ref. 50979254, Qiagen), according to the manufacturer’s instructions. By the use of a spectrophotometer, RNA concentration was determined (260 and 280 nm) and RNA quality assessed by visualization of 28S and 18S rRNA bands, after electrophoresis through a 1.5% gel agarose and ethidium bromide staining. Reverse transcription was carried out using Reverse Transcriptase Superscript III enzyme (ref. 18080093, Invitrogene, Gibco, Carlsbad, CA, USA), from 1 mg total RNA in a 20 mL reaction volume, using oligo (dT) primer (27–7858-01, GE Healthcare, Buckinghamshire, UK). Different Internet-based interfaces, such as Primer-3 (Untergasser et al. 2012) and Primer Premier Software (Premier Biosoft Int., Palo Alto, CA, USA) were used for specific primers for target genes. Several conventional PCR reactions were carried out using a default thermocycler (Applied Biosystems, Foster City, CA, USA) as follows:

2 min at 94 °C for denaturation; 35 cycles of 15 s at 94 °C for enzyme activation, 45 s at 57–60 °C for annealing (depending on the gene-*PGR*-57.8 °C; *ESR2*-58.5 °C and *ESR1*-60 °C) and 45 s at 68 °C for extension; and 5 min at 68 °C for finalization. The design of all primers for two different exons followed specific guidelines in order to avoid genomic DNA amplification (www.qiagen.com). All reactions were carried out in duplicate in 0.2-mL PCR tubes (PCR-0.2-C, Axygen 321-02-051, Corning, CA, USA) in a 25 μL reaction volume: 8.5 μL water; 1 μL forward primer (10 pmol/μL); 1 μL reverse primer (10 pmol/μL); 12.5 μL using FideliTaq DNA polymerase master mix (71180, USB, Cleveland, OH, USA), and 2 μL of cDNA. All Agarose (2%) (BIO-41025, Bioline, Luckenwalde, Germany) electrophoresis gel and ethidium bromide (17896, Thermo, Waltham, MA, USA) staining showed a specific and single product. For dissociation curve analysis, cDNA was amplified with real-time PCR, as describe before. All primers were validated and used at 80 nM.

### 4.6. Progesterone Analysis

Evaluation of progesterone concentration was in plasma using a solid-phase radioimmunoassay (Coat-a-Count Progesterone, Diagnostic Product Corp., Los Angeles, CA, USA). Intra-assay coefficient was 6.4% for the level of 3.2 nmol/L (1 ng/mL) and 4.2% for the level of 15.9 nmol/L (5 ng/mL).

### 4.7. Chemical Studies

The total content of the inguinal sinus was collected from non-pregnant (NP), pregnant (P), and post-parturient (PP) ewes (*n* = 28) and extracted with ethyl acetate using an ultrasonic bath (1 mg/mL; 5 min). The total content of the inguinal sinus was collected from non-pregnant (NP), pregnant (P) and post parturient (PP) ewes (*n* = 28) and extracted with ethyl acetate using an ultrasonic bath (1 mg/mL; 5min). After, centrifugation (2000 rpm/15min), the supernatant was evaporated to dryness under vacuum at a temperature below 40 °C. The obtained residue was dissolved in ethyl acetate (1 mL) and then analyzed by thin layer chromatography (TLC) using different chromatographic and derivatization systems, including silica gel F254 Merck as stationary phase, and toluene: ethyl acetate (9:1 *v*/*v*) as mobile phase and Liebermann and Dragendorff as spraying reagents [83]. After derivatization, visible and UV light at 365 nm used for data acquisition. Samples of the surrounding skin prepared in the same way used as negative control.

### 4.8. Statistical Analysis

Flow cytometry data of ERSR1, ESR2, PRLR, and PGR proteins in IGS from ewes in the follicular, luteal, pregnancy, and post-partum phases, were subjected to a one-way analysis of variance (ANOVA). Significance was defined as values of *p* < 0.05. For statistically different results, the means were further analyzed by post hoc comparison test, such as LSD (least significant differences) and Scheffé tests (probabilities for post hoc tests).

## 5. Conclusions

Our data, to the best of our knowledge, have not been reported elsewhere. These findings concern the expression of steroid hormones and prolactin receptors in sheep IGS and point out that a particular triterpenoid-rich secretion and organic nitrogen compounds that totally differ from the surrounding skin are present in IGS. These secretions show modifications, particularly during pregnancy and post-partum. Altogether, IGS secretion in the ewe might contribute to the putative involvement of this gland in the signaling cues that direct the lamb to the mammary gland.

Since impairment of bond formation between ewes and their offspring is a common cause of offspring death, this research will potentially benefit farmers by contributing to the increased survival of lambs due to understanding and using these odor products. As stated, IGS fluctuations in the expression of ERS1, ERS2, PGR, and PRLR are present and might be linked to putative chemosignals. Further research by our team will give rise to new data regarding a direct hormonal cause/effect on IGS, in agreement with other researchers who claim to have achieved methods of manipulating reproduction in domestic ungulates by using specific odors.

## Figures and Tables

**Table 1:** Specific primers were designed—sequences used for quantitative real-time PCR (bp = base pair).

Gene (Acession Number)	Sequence 5′–3′ Amplicon (Base Pairs)
*ESR1* (XM_015097472.1)	Forward: CCATGGAATCTGCCAAGGAG (167 bp) Reverse: ATCAATTGTGCACTGGTTGGT
*ESR2* (NM_001009737.1)	Forward: TGGAGTCTGGTCATGTGAAGGA (150 bp) Reverse: TCATAGCACTTCCGCAGTCG
*PGR* (XM_015100878.1)	Forward: CAGCCAGAGCCCACAGTACA (176 bp) Reverse: TGCAATCGTTTCTTCCAGCA
*PRLR* (NM_001009204.1)	Forward: GTCTCCACCCACCCTGACTG (320 bp) Reverse: AAGCCACTGCCCAGACCATA

## Supplementary Materials

Supplementary materials can be found at www.mdpi.com/1422-0067/18/7/1516/s1.
