# Supplementary figures and images for: Understanding the Inguinal Sinus in Sheep (Ovis aries)—Morphology, Secretion, and Expression of Progesterone, Estrogens, and Prolactin Receptors
Source: Int J Mol Sci. 2017 Jul 13;18(7):1516. doi: 10.3390/ijms18071516 (PMC5536006; doi:10.3390/ijms18071516)

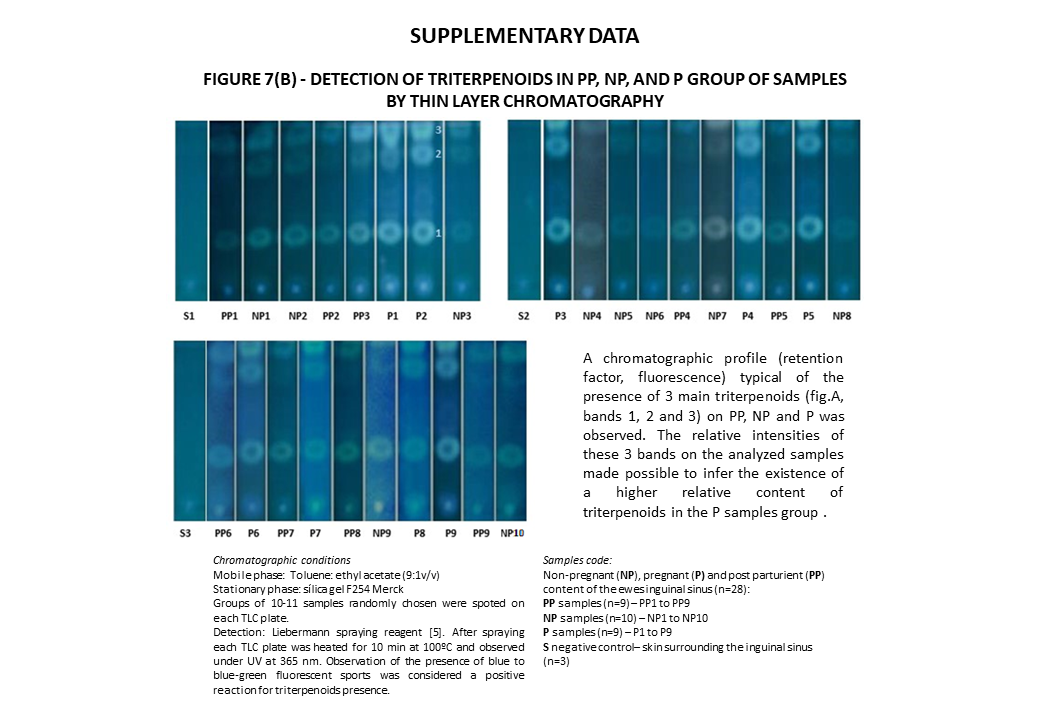

Supplement: Supplementary file 1 [file ijms-18-01516-s001.zip › ijms-197045-Supplementary Data.tif]
